# A Model Falsification Approach to Learning in Non-Stationary Environments for Experimental Design

**DOI:** 10.1038/s41598-019-54145-7

**Published:** 2019-11-29

**Authors:** Andrea Murari, Michele Lungaroni, Emmanuele Peluso, Teddy Craciunescu, Michela Gelfusa

**Affiliations:** 1 Consorzio RFX (CNR, ENEA, INFN, Universita’ Padova, Acciaierie Venete SpA), Corso Stati Uniti 4, 35127 Padova, Italy; 2 Department of Industrial Engineering, University of Rome “Tor Vergata”, via del Politecnico 1, 00100 Rome, Italy; 3 National Institute for Laser, Plasma and Radiation Physics, Atomistilor Street 409, Magurele-Bucharest, RO-077125 Romania

**Keywords:** Information technology, Software

## Abstract

The application of data driven machine learning and advanced statistical tools to complex physics experiments, such as Magnetic Confinement Nuclear Fusion, can be problematic, due the varying conditions of the systems to be studied. In particular, new experiments have to be planned in unexplored regions of the operational space. As a consequence, care must be taken because the input quantities used to train and test the performance of the analysis tools are not necessarily sampled by the same probability distribution as in the final applications. The regressors and dependent variables cannot therefore be assumed to verify the i.i.d. (independent and identical distribution) hypothesis and learning has therefore to take place under non stationary conditions. In the present paper, a new data driven methodology is proposed to guide planning of experiments, to explore the operational space and to optimise performance. The approach is based on the falsification of existing models. The deployment of Symbolic Regression via Genetic Programming to the available data is used to identify a set of candidate models, using the method of the Pareto Frontier. The confidence intervals for the predictions of such models are then used to find the best region of the parameter space for their falsification, where the next set of experiments can be most profitably carried out. Extensive numerical tests and applications to the scaling laws in Tokamaks prove the viability of the proposed methodology.

## Introduction

## Experimental design in non stationary environments

In the last two decades, incessant technological developments and various breakthroughs in computer and automation have led to an increased complexity of the data to be processed, very often in real time. This complexity is also linked to the fact that nowadays automated tools are expected to monitor and control systems, whose dynamics can change due to their internal evolution or external disturbances. The consequent variations in the systems require sophisticated forms of adaptive learning under “concept shift^1^”.

The problems posed by non-stationary conditions are particularly relevant in many scientific applications, especially for the planning of experiments and the design of new devices. These tasks inherently require extrapolation to unchartered territory. Indeed, when planning new experiments, it is essential to explore new regions of the operational space, to increase knowledge as much as possible. These issues, of planning new experiments and designing new devices, are particularly challenging in the case of complex, nonlinear systems difficult to model from first principles. For such systems, the only alternative consists of deriving empirical models directly from the data, using some forms of data mining such as genetic programming and machine learning. The non-stationarity of the phenomena, whose behavior the models have to predict, is often neglected but should not be underestimated, because the training set can be statistically quite different from the test set and the final application. On the contrary, the mathematics of practically all statistical and machine learning tools hinges on the i.i.d. (independent and identical distribution) assumption. The i.i.d conditions imply that the data are sampled independently from a stationary distribution function, which must be the same for the training set, for the test set and for the final deployment conditions. In many experiments in physics and chemistry, the i.i.d assumption is strongly violated, resulting in suboptimal performance of the models and predictors. This is certainly true for example in Magnetic Confinement Nuclear Fusion (MNCF). In MNCF the experiments, called discharges, are pulsed. On JET, the largest Tokamak in the world, they can last for tens of seconds and they can be repeated about every half an hour^2–5^. The parameter settings of each discharge are typically different, depending on the results of the previous experiments. The i.i.d. conditions are therefore violated and the models to plan future experiments have to be derived under conditions of “concept shift”.

Contrary to other approaches, the methodology presented in the following does not make any “a priori” assumption about the probability density functions (pdf) of the regressors; on the contrary it helps determining their most appropriate values for performing new experiments. On the other hand, it is predicated on the hypothesis that the concept shift is slow and therefore that experiments can be planned on the basis of previous data, which carry extrapolable information on the status of the system under investigation. To address the issue of slow “concept shift”, the models are derived using Symbolic Regression (SR) via Genetic Programming (GP)^6,7^. The choice of this family of techniques is driven by the consideration that they can typically output more scientific meaningful equations than traditional machine learning methods. With this very powerful tools, a series of candidate models are selected, using the approach of the Pareto Frontier. Nonlinear fitting of the data allows determining the confidence intervals of the candidate models^8^. At this point, a numerical exploration of the operational space permits to identify the best combination of parameters to discriminate between the models. This typically means selecting a combination of the independent variables, for which the predictions of the models are different well outside their confidence intervals. Once the most advantageous operational region to investigate is selected, experiments are performed, new data are collected and the process can be repeated. SR via GP can be deployed again to identify a new set of candidate models to plan additional experiments. The process ends when only a single model, or a sufficiently limited number of candidate models, has been identified.

The paper is structured as follows. The technique of Symbolic Regression via Genetic Programming is briefly reviewed in the next Section. The description of the proposed method of learning in non-stationary conditions for experimental design is the subject of Section 3, together with examples of the numerical tests performed to verify the viability of the approach. A real life application, the design of experiments to investigate the scaling laws for the confinement time in Tokamaks, using the data of an international multimachine database, is discussed in Section 4. Discussion and future developments are the subject of the last Section 5.

## Symbolic Regression via Genetic Programming for Data Driven Modelling

**Figure 1:**
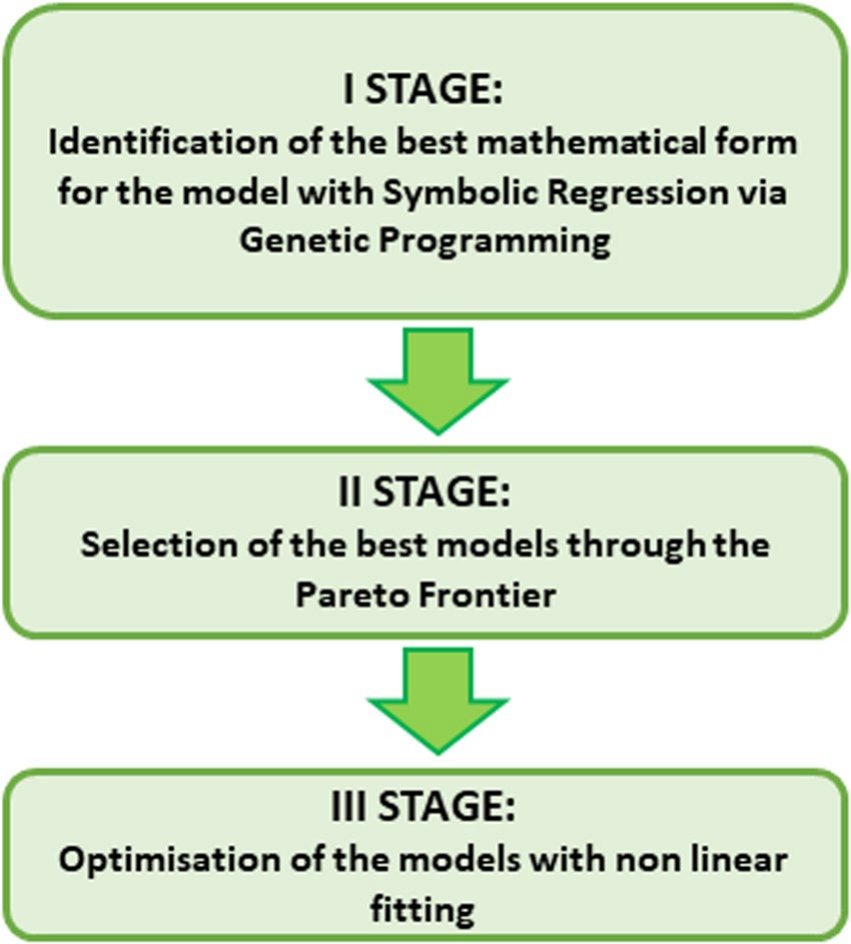
Block diagram of the steps required to perform Symbolic Regression via Genetic Programming for the data driven derivation of mathematical models.

As mentioned in the introductory section, this paper presents a new methodology for experimental design. In this perspective, the main tool used is Symbolic regression via Genetic Programming. The technique consists basically of testing a large number of mathematical expressions to fit a given database. To keep the number of alternatives to be evaluated at a manageable level, various generations of models are built. The most performing ones of the previous generation are retained and used as the basis for producing a new generation of models with genetic programming techniques. The flow chart showing the main steps of the methodology is reported in Fig. 1.

In more detail, in terms of knowledge representation, the various candidate formulas are expressed as trees. In this context, the trees can be considered as consisting of functions and terminal nodes. The functional nodes can be arithmetic operators, any type of mathematical functions and saturation terms^6,7^. This representation of the formulas is not unique but it is the preferred one because it permits an easy implementation of Genetic Programming (GP) operations. Genetic Programs are computational techniques, which have been explicitly developed to solve complex optimization problems, mimicking the genetic processes of living organisms. They operate on a population of individuals, e.g mathematical expressions in our application. Each individual represents a possible solution, a potential model of the phenomenon under investigation in our case. One of the crucial aspects of SR via GP is the qualification of these candidate models. Such an evaluation is based on specific indicators called fitness functions (FFs). The FF is selected to measure how good an individual is with respect to the database. Once the best individuals have been identified, on the basis of the FF, genetic operators (Reproduction, Crossover and Mutation) are applied to them to obtain the new population. Therefore SR via GP operates in such a way that better individuals are more likely to have more descendants than inferior individuals. The iteration is stopped when a stable and acceptable solution is identified. At this point, the algorithm provides the solution with best performance in terms of the FF^9–12^.

The fitness function is probably the most crucial element of the genetic programming approach, because it determines the quality of the candidate solutions. Various indicators have been used in the past to implement the FF: the Akaike Information Criterion (AIC), the Takeuchi Information Criterion (TIC) and Bayesian Information Criterion (BIC)^13,14^. It is worth mentioning that, in all numerical and experimental cases reported in the paper, BIC and TIC have provided the same results as AIC, which is discussed in more detail in the following as a representative example. The AIC is explicitly conceived to reduce the generalization error. Indeed it can be demonstrated that AIC provides an unbiased estimate of the predictive inaccuracy of a model. The most widely used form is:

1 $$AIC=n\cdot \,\mathrm{ln}(\frac{RMSE}{n})+2k$$

where RMSE is the Root Mean Square Error, errors indicate the residuals, the difference between the experimental values and the estimates of the scaling laws, *k* is the number of nodes in the model and *n* the number of entries in the database.

The AIC and the other mentioned criteria are indicators to be minimised, in the sense that better models have lower values. This property can be appreciated by inspection of the various indicators. Indeed, all of them consist basically of two parts. The first one depends on the quality of the fit (on the RMSE of the residuals for the case of the AIC). Models closer to the data have lower values of this term. The second addend implements a penalty for complexity, since it is proportional to the number of nodes in the trees representing the model equations. All the mathematical details to fully appreciate the relative merits of these criteria can be found in^14^.

**Figure 2:**
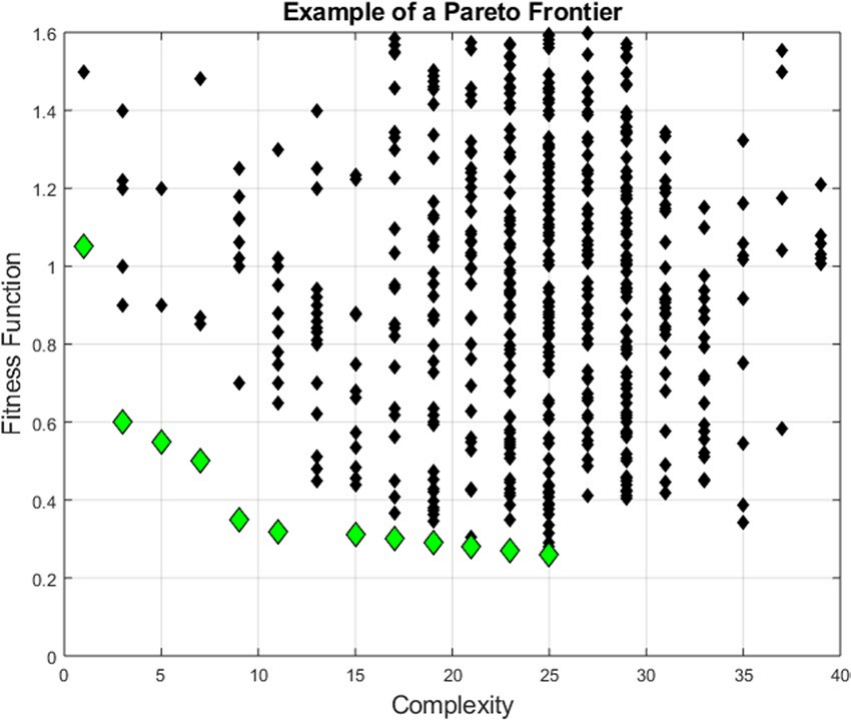
The models indicated with diamonds form the Pareto Frontier, since they have the best values of the FF for each level of complexity.

In practical applications, given the limitations of the databases available, typically the Fitness Functions does not manage to converge on a single individual model, clearly outperforming all the others. Normally, SR via GP provides a series of models, which are good candidates for the interpretation of the data available. The main tool implemented, to select the final candidate models, is the Pareto Frontier (an example is shown in Fig. 2). The Pareto Frontier (PF) reports the best model for each level of complexity, according to the FF. As can be appreciated from Fig. 2, the PF presents an L shape, meaning that there is a tendency of lower returns: increasing the complexity above a certain level does not produce significant improvements in the fitting quality of the models. The models around the inflexion points of the PF are the ones, which require attention and are the best candidates for extrapolation.

The last step of the methodology consists of nonlinear fitting of the candidate models, identified with SR via GP^8^. This is an essential phase to associate confidence levels to the estimates of the models, which is an indispensable piece of information for scientific applications.

## Exploring the Operational Space with SR via GP

In this paper, a new alternative is proposed to complement the traditional approaches of training under concept shift. The main motivation is that, in the planning of future experiments, the pdf of the new experiments is unknown and the conditional probabilities of the dependent variables on the regressors are not necessarily the same. Therefore, the proposed technique is based on the selection of the parameter range more appropriate for the falsification of the available models. The database of past experiments is analyzed first with SR via GP. The main idea consists then of selecting the reasonable candidate models on the basis of the Pareto Frontier. After application of the nonlinear fitting procedures, it is possible to associate confidence intervals to the predictions of the models. With this information available, the developed algorithm traverses the unexplored operational space to identify the regions closest to the past data, where the predictions of the candidate models differ outside the confidence intervals. In other words, the technique determines the smallest variations in the operational parameters of the experiments to falsify the derived models. The range of parameters closest to the one already explored is selected for two main reasons: a) because typically this is the most accessible region for additional experiments b) because predictions of the previous models in this region are expected to be the most robust even in presence of concept shift. Once the experiments have explored the new region of the operational space and new data are collected, the process can be repeated until convergence on a sufficiently specific model for the interpretation of the phenomena under study.

The methodology just described has been subjected to a systematic series of numerical tests. Many families of mathematical functions have been used to generate synthetic data. Various levels of Gaussian noise have been added to the points generated by the numerical functions to simulate experimental conditions. The proposed method has then been iteratively applied to the data until the original function is identified. The results have always been positive and the proposed technique has always allowed recovering the original equations generating the data in a very efficient way. In the following, a quite challenging example is described in some detail to show the potential of the proposed approach.

For clarity’s sake, a low dimensional case is illustrated, but it has been verified that the approach is equally valid for high dimensional cases, provided of course a sufficient number of good quality examples and adequate computational resources are available.

**Figure 3:**
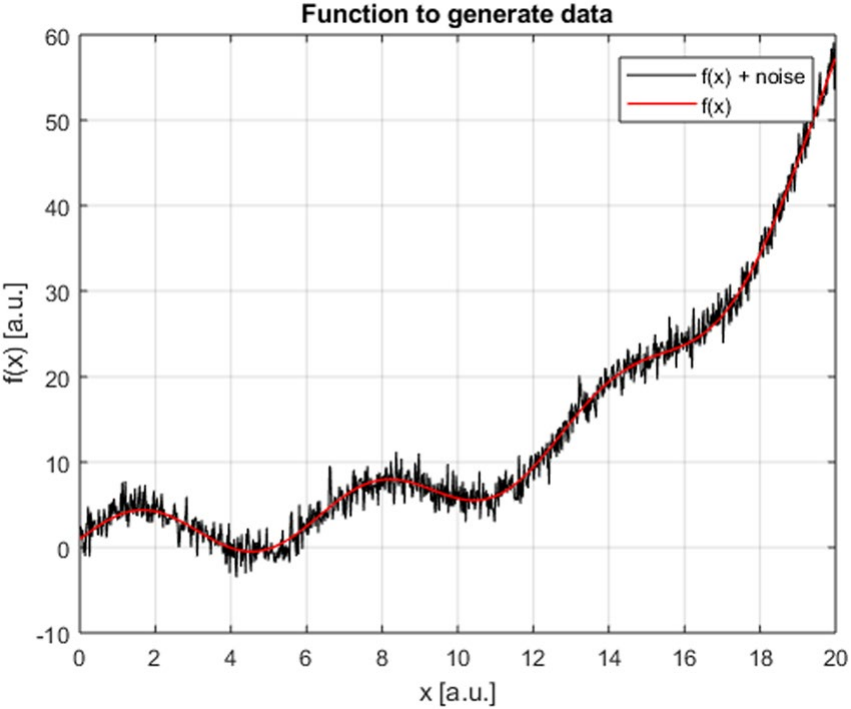
Function of one variable to illustrate the methodology. The red line is the plot of the function generating the data, Eq. (2). The black line reports the actual data including the noise.

In Fig. 3, a simple example of a function depending on a single regressors is shown. The equation of the function used to generate the data is:

2 $$f(x)=3\,\sin \,x+\exp (x/5)$$

Gaussian noise, of zero mean and variance equal to 10% of the dependent variable absolute value, has been added to the individual points.

The first training has been performed by generating 100 points in the interval between 0 and 2. The best three solutions identified from the Pareto Frontier, after application of SR via GP, are:

$$\begin{array}{c}{y}_{1,1}=2.28(\sin \,x+\frac{1}{1+exp(-1.26x)})\\ {y}_{2,1}=4.38\,\sin \,{x}^{0.62}\\ {y}_{3,1}=3.53{x}^{0.41}\end{array}$$

**Figure 4:**
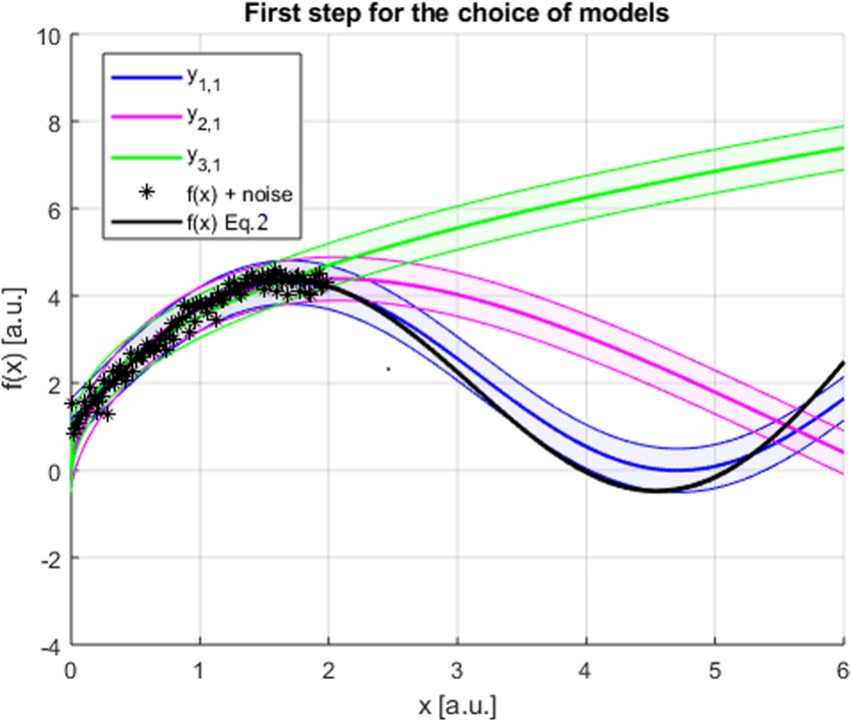
First step to converge on the final and correct solution. The data provided to SR via GP are 100 points in the in the interval between 0 and 2. The three best candidates derived from the Pareto Frontier are reported in green, red and blue with the relative confidence intervals. The input points are depicted in black. In black also the actual function generating the data.

The three functions are shown Fig. 4; from the confidence intervals, it appears clearly that a very advantageous range of *x* to falsify the three solutions is the one between 3 and 5. Synthetic points have therefore been generated in that interval simulating the results of additional experiments. SR via GP has then been applied including this new data to obtain additional candidate models. By repeating the process two more times, it has been possible to converge on the final set of equations:

$$\begin{array}{c}{y}_{1,4}=3\,\sin \,x+\exp (0.2x)\\ {y}_{2,4}=3.40\,\sin \,x+0.0073\,{x}^{2.96}\\ {y}_{3,4}=0.00007\,{x}^{4.48}+2\end{array}$$

**Figure 5:**
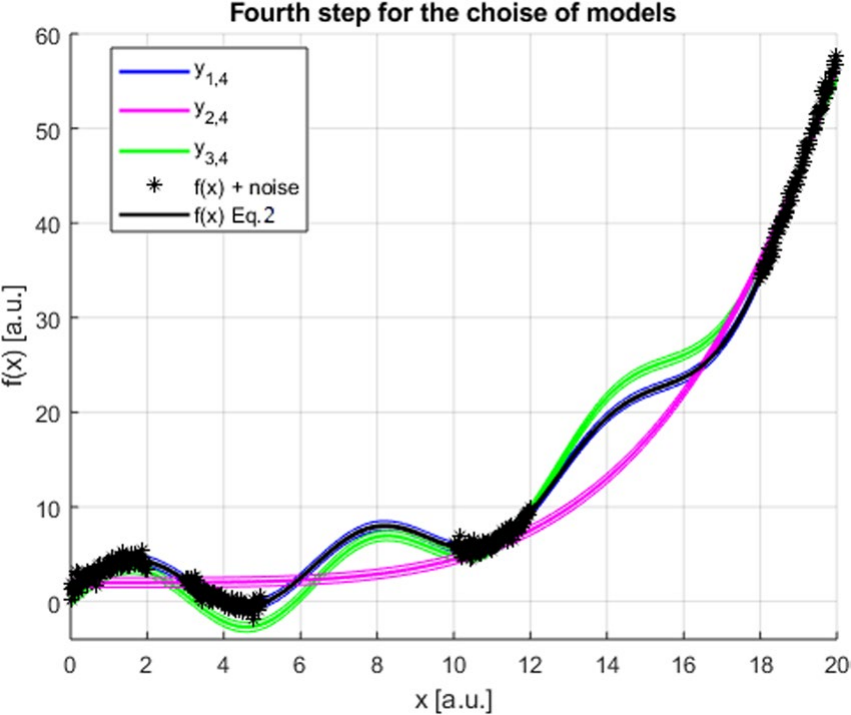
Third and final step to converge on the final and correct solution. The new data provided to SR via GP are 100 points in the interval in the interval between 16 and 18 (after two previous iteration with data between 3 and 5 and between 10 and 12). The three best candidates derived from the Pareto Front are reported in green, red and blue with the relative confidence intervals. The input points are depicted in black. In black also the actual function generating the data.

The three candidate equations are plotted in Fig. 5. They all fit the input data acceptably but now the right solution can be clearly identified because it outperforms significantly all the others in terms of all statistical indicators. It should be mentioned that traditional methods of covariate shift^15^, to analyse the data in non-stationary conditions, would be significantly less efficient in solving the problem and converging on an acceptable solution, because they have problems handling the oscillating character of the function generating the data.

## Real World Example: Scaling of Confinement Time in Tokamaks

As an example of real world applications, in this section the case of scaling laws is discussed. Scaling laws are particularly important not only for the understanding of the physics but also for the planning of new experiments and devices. In the Tokamak community, the extrapolation of the energy confinement time to the next generation of machines has been studied for several decades. Unfortunately, the problem is too complex to be attacked with models based on first principles. Therefore, various empirical scaling expressions have been proposed based on the analysis of data collected in previous experiments. Their extrapolation to future experiments and devices is a typical example of learning under covariate shift. Since the objective is to apply the derived models to larger machines, and therefore in a completely different range of parameters, they are typically predicated on the assumption that the scalings are in power law form. To show the potential of the proposed approach, the devised method of learning under covariate shift is tested using an international experimental database for the confinement time in Tokamaks, DB3v13f^16^. This database was explicitly conceived to support advanced studies of the confinement time, including validated signals from most relevant Tokamak machines ever operated in the world. In line with the previous literature on the subject, the following quantities have been considered good candidate regressors in the present work:

$$B[T],I[MA],n[{10}^{19}{m}^{-3}],\,R[m],\varepsilon ,k,P[MW]$$

**Figure 6:**
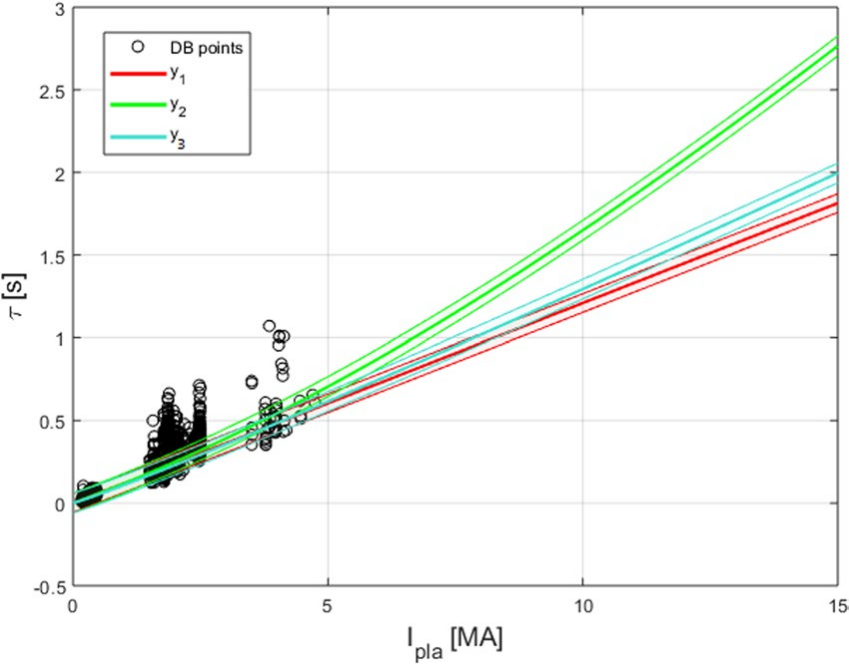
The three intervals of plasma current (x axis) sampled to identify the final scaling laws for the energy confinement time (y axis) reported in Table 1: below 0.5 MA, between 1.5 and 2.5 MA and between 3.5 and 5 MA. Again the black dots indicate the experimental data used as input to the three iterations of the procedure.

In the previous list, *k* indicates the volume elongation, *ε* the inverse aspect ratio, n the central line average plasma density, B the toroidal magnetic field, R the plasma major radius, I the plasma current and finally P the estimated power losses. The main objective of the analysis is to identify the best range of plasma currents to obtain a good confinement. To exemplify the potential of the proposed approach, SR via GP has been applied first to the smaller devices in the database, i.e. all the devices except JET, which operated at a plasma current up to 0.5 MA. Two iterations of the proposed procedures identified as optimal the current intervals between 1.5 and 2.5 MA and between 3.5 and 5 MA to collect new data, as shown in Fig. 6.

Including the data of experiments carried out in these intervals allows reducing the candidate models to three main families of equations, reported in the following:

$$\begin{array}{c}{y}_{1}=0.076\cdot I\cdot {R}^{2}\cdot k\cdot {P}^{-1}\cdot \frac{1}{1+exp(-0.201{n}^{2.004})}\cdot {(\frac{1}{n\cdot {P}^{1.293}}\cdot \frac{1}{1+exp(0.715M)})}^{-0.1761}\\ {y}_{2}=0.1831\cdot I\cdot {R}^{2}\cdot {P}^{-0.662}\cdot \frac{1}{1+exp(-0.094\cdot I)}\cdot \frac{1}{1+exp(-0.408\cdot n)}\\ {y}_{3}=0.070\cdot {I}^{1.071}\cdot {R}^{1.706}\cdot {k}^{1.250}\cdot {P}^{-0.715}{n}^{0.100}\cdot \frac{1}{1+exp(-0.408\cdot {n}^{1.036})}\end{array}$$

The new equations narrow down the extrapolations to the next international device, ITER, to a reasonable interval between 4.35 and 2.85 s (see Table 1). Also ITER plasma current, 15 MA, seems to be more than adequate to discriminate between even the closest scaling laws *y*_2_ and *y*_3_ (see Fig. 6).

**Table 1:** The prediction for the confinement time of ITER for the three families of scaling laws identified after the third iteration of the proposed procedure.

Model	τ_ITER_
*y*_1_	2.55 s
*y*_2_	4.34 s
*y*_3_	2.85 s

It is important to notice that the equations, derived with the proposed approach, are practically the same as the ones already extracted using the entire ITPA database but have been obtained using only about 50% of the entries^10^. Of course, a reduction of such a factor in the experimental investments and efforts would be extremely significant, given the cost of running present day large fusion devices.

## Conclusions

In this paper, an original methodology is presented to guide scientists in the design of experiments in new regions of the operational space. This is a very important step in the scientific process, since only exploration of new regions of the parameters space can provide real new information and knowledge. On the other hand, planning experiments in an unexplored range of parameters is delicate both conceptually and practically, because the extrapolation of previous knowledge is uncertain. Since the available models have been derived in conditions different from the ones of the final applications, the i.i.d assumptions cannot be invoked. Consequently, care must be taken because the models obtained with old data can perform sub optimally and provide even wrong answers.

To alleviate this problem, the methodology proposed in the paper is based on the falsification of the models with experiments in the range of parameters as close as possible to the previous experiments. The approach, based on SR via GP, has been successfully tested with a variety of numerical tests. The application to a multimachine international database of Tokamak devices has also provided very encouraging results. It is worth mentioning that practically the same methodology can be adopted to fine tune the analysis of existing databases. In various scientific fields, large data sets are indeed available but only a small subset of the entries can be analysed in detail due to the lack of man power. The suggested approach, of falsifying the models obtained with SR via GP, can also provide clear indications about the most important entries, on which to concentrate further investigations. On the other hand, the proposed methodologies can help only addressing “soft nonlinearities” and not catastrophic events, not having left any signature in the available data. This is of course a limitation of all data driven techniques, which are designed to learn what is in the data and cannot provide guidance about information not available in their inputs. Nonetheless it should be considered that the proposed approach of SR via GP can be advantageous, compared to other methodologies, at least in two respects. First, by better modelling soft nonlinearities, they can help in approaching regions at risk of catastrophic changes. Moreover, if “a priori” information about the nature and the possible localisation in the parameter space of strong nonlinearities is available, the models can be steered to favour solutions including this knowledge (for example by selecting appropriate basis functions).

With regard to future developments, more sophisticated forms of the fitness function, such as the Widely Applicable Information Criteria^17^ and Generalised information criteria^18^, can be certainly implemented without any particular conceptual difficulty, provided of course the quality and quantity of the data and the computational resources are adequate to take advantage of them. Another important topic to be further refined is the treatment of the errors. Methods of Information Geometry, particularly the Geodesic Distance between probability density functions, are expected to have strong potential to improve the capability of the proposed methodology^19^. On a longer time scale, it should be remembered that the proposed techniques are purely statistical. Of course, the availability of actual causal models would improve the confidence in the design of new experiments and/or machines. Identifying good candidate causal models from databases is therefore one of the main lines of research for the future research on learning under systemic concept shift. Comparing the results of the proposed tools with Graphical and Bayesian Network Models^20,21^ is also expected to generate very interesting new insights.

## Data Availability

The ITPA database (DB3v13f) used for this study: https://urldefense.proofpoint.com/v2/url?u=http-3A__efdasql.ipp.mpg.de_threshold_Public_ThresholdDbInfo_ThresholdvarDB2-5F3.htm&d=DwIBaQ&c=vh6FgFnduejNhPPD0fl_yRaSfZy8CWbWnIf4XJhSqx8&r=W8RE-88OJ6HkLx5-vnSwGMbECZfnybKmwCXImBggWgpae5ASEwQeYcLKZnw2tz-i&m=gnXLSpcZeP2D39tup-cwv45ohkSr48CVu0tlFO2UmW4&s=MtDspa 2m7fXrriEAMABTQ5R6ElmLaECxYmdho4lzD_c&e=. If the link is offline, contact the corresponding author.
